# Evaluation of the sensitization rates and identification of IgE-binding components in wild and genetically modified potatoes in patients with allergic disorders

**DOI:** 10.1186/1476-7961-4-10

**Published:** 2006-07-04

**Authors:** Soo-Keol Lee, Young-Min Ye, Sung-Ho Yoon, Bou-Oung Lee, Seung-Hyun Kim, Hae-Sim Park

**Affiliations:** 1 Department of Internal Medicine, College of Medicine, Dong-A University, Busan; 2 Department of Allergy & Rheumatology, School of Medicine, Ajou University, Suwon; 3 College of Agriculture, Chonbuk National University, Chonju, Korea

## Abstract

**Background:**

The potato is one of the most common types of genetically modified (GM) food. However, there are no published data evaluating the impact of genetic manipulations on the allergenicity of GM potatoes. To compare the allergenicity of GM potatoes with that of wild-type potatoes using *in vivo *and *in vitro *methods in adult allergy patients sensitized to potatoes.

**Methods:**

A total of 1886 patients with various allergic diseases and 38 healthy controls participated in the study. Skin-prick testing and IgE-ELISA were carried out with extracts prepared from wild-type and GM potatoes. An ELISA inhibition test was used to confirm the binding specificity. IgE-binding components in extracts from the two types of potato were identified by SDS-PAGE and IgE-immunoblotting. The effects of digestive enzymes and heat on the allergenicity of the extracts was evaluated by preincubating the potatoes with or without simulated gastric and intestinal fluids in the absence or presence of heat.

**Results:**

Positive responses (ratio of the wheal size induced by the allergen to that induced by histamine (A/H) ≥ 2+) to wild-type or GM potato extracts, as demonstrated by the skin-prick test, were observed in 108 patients (5.7%). Serum-specific IgE was detected in 0–88% of subjects who tested positively. ELISA inhibition tests indicated significant inhibition when extract from each type of potato was added. IgE-immunoblot analysis demonstrated the presence of 14 IgE-binding components within the wild-type potato and 9 within the GM potato. Furthermore, a common 45-kDa binding component that yielded similar IgE-binding patterns was noted in more than 80% of the reactions using sera from patients sensitized to wild-type or GM potato. Exposure to simulated gastric fluid and heat treatment similarly inhibited IgE binding by extracts from wild-type and GM potatoes, whereas minimal changes were obtained following exposure of the extracts to simulated intestinal fluid.

**Conclusion:**

Our results strongly suggest that genetic manipulation of potatoes does not increase their allergenic risk. The sensitization rate of adult allergy patients to both types of extract was 5.7%, and a common major allergen (45 kDa) was identified.

## Background

Food-induced allergic reactions are responsible for a variety of symptoms involving the skin, gastrointestinal tract, and respiratory tract, and proceed through IgE- and non-IgE-mediated mechanisms. The major foods responsible for food-induced allergic reactions are milk, eggs, peanuts, fish, and tree nuts in children, and peanuts, tree nuts, fish, and shellfish in adults [1]. There are some reports in the literature on allergic reactions to potatoes; for example, a child developed urticaria and angioedema after eating a potato [2,3], bronchial asthma was induced in an individual while peeling a raw potato [4], and anaphylaxis developed in response to raw potatoes [5]. Furthermore, potatoes have been found to cross-react with birch pollen, fruits, and latex [6,7].

Agricultural biotechnology has tremendous implications for both agriculture and the general public. Insect-resistant corn and herbicide-tolerant soybeans are grown on 30–50% of the total acreage planted with these crops in North America [8]. Previous studies comparing the allergenicity of wild-type and genetically modified (GM) corn demonstrated that the allergic risk was not increased after genetic manipulation [9-11]. In Korea, potato, soybean, and corn are the most commonly exposed GM foods; however, to date, there are no reports on the allergenic risk of GM potatoes.

In this study, the sensitization rates of adult allergy patients in response to wild-type and GM potatoes were evaluated by skin-prick test and ELISA (enzyme linked immunosorbent assay). SDS-PAGE (sodium dodecyl sulfate-polyacrylamide gel electrophoresis) and IgE-immunoblotting were carried out to identify the major allergens present in the potato extracts. To evaluate the effects of digestive enzymes and heat on the allergenicity of the two types of potato, the extracts were preincubated with or without simulated gastric and intestinal fluids in the presence or absence of heat.

## Methods

### Subjects

Sensitization rates to the two potato extracts were evaluated in 1886 allergy patients and in 38 healthy non-atopic subjects. The participants, who ranged in age from 15 to 65 years, were enrolled in the study by the Department of Allergy and Rheumatology, Ajou University School of Medicine, Suwon, Korea. The GM potato, carrying the neomycin phosphotransferase II (NPTII) and phosphinothricin acetyltransferase (PAT) genes (Table 1), was provided by ChonBuk National University, Chunju, Korea. The wild-type potato was produced in Korea. From January 2004 to October 2004, 1886 patients admitted to the hospital for the treatment of various allergic diseases, including asthma, allergic rhinitis, and food and drug allergy, were skin-prick tested with common inhalant allergens and with extracts from GM and wild-type potatoes. In the skin-prick tests, 50 common inhalant allergens, 30 food allergens, and the potato extracts were applied using 26-gauge needles to the backs of the patients. The results were read 15 min later. A positive reaction was defined as a mean wheal diameter of ≥3 mm. The size of the wheal produced by each antigen or by the positive control histamine was expressed in terms of maximum diameter and vertical length at the midportion of the maximal length. Skin reactivity was expressed as the ratio of the wheal size induced by the allergen and that induced by histamine (A/H). The data were recorded in accordance with the recommendations of the Standardization Committee of the Northern Society of Allergology [12]. An A/H of 0.1–1 and an erythema diameter of <21 mm was assigned a reactivity of 1+. An A/H of 0.1–1 and erythema >21 mm was assigned a reactivity of 2+. An A/H in the range of 1–2 was recorded as a reactivity of 3+. For an A/H of 2–3, reactivity was 4+, and for an A/H >3 reactivity was 5+. A positive responder was defined as a subject who demonstrated a response >2+ on the skin-prick test. This study was reviewed by the institutional review board of Ajou University Medical Center, Suwon, Korea.

**Table 1:** Primer sequences of inserted PAT and NPT genes.

	Primer sequence
PAT 2587U	5'-TCG TCA ACC ACT ACA TCG AGA-3
PAT 2893L	5'-ATG ACA GCG ACC ACG CTC TT-3'
NBT 3161U	5'-AGA AAG TAT CCA TCA TGG CTG A-3'
NBT 3574L	5'-ATA CCG TAA AGC ACG AGG AAG-3'

### Preparation of extracts from wild-type and GM potatoes

Extracts were prepared from wild-type and GM potatoes using phosphate-buffered saline (PBS; pH 7.5, 1:10 w/v), kept at 4°C overnight, and then centrifuged at 12,000–15,000 rpm for 20 min. The supernatant was dialyzed (the cutoff molecular weight was 6000 Da; Spectrum Medical Industries, Houston, TX, USA) against 4 l of PBS at 4°C for 72 h, and the resultant fluid was stored at -20°C until ELISA, ELISA inhibition testing, and immunoblot analyses were carried out. For the skin-prick test, extract was mixed with sterile glycerin at a ratio of 1:1. To evaluate the effects of digestive enzymes, simulated gastric fluid (SGF; 471 U pepsin/mg; Sigma Chemical Co., St. Louis, MO, USA) and simulated intestinal fluid (SIF; pancreatin; Sigma) were preincubated with the extracts in the presence or absence of heat.

### ELISA for specific IgE antibodies to potato extracts

The presence of specific IgE antibodies to the two types of extract was determined by ELISA, as previously described [13]. Microtiter plates (Corning, NY) were coated with 100 μl of extract (GM or wild-type, 10 μg/ml)/well and kept overnight at 4°C. Each well was washed three times with 0.05% PBS-Tween (PBST), and the remaining binding sites were blocked by incubation with 10% fetal bovine serum (FBS)-PBS for 1 h at room temperature. The wells were then incubated for 2 h at room temperature with either 50 μl of the patients' sera or the control sera, both at 50% dilution. The control sera were from the 38 healthy controls who had tested negative in the skin-prick tests to the common inhalant and food allergens and to potatoes. The wells were washed three times with PBST, 1:1000 v/v biotin-labeled goat anti-human IgE antibody (Vector Lab, Burlingame, CA, USA) was added to each well, and the plates were incubated for 1 h at room temperature. After another washing, 100 μl of 1:1000 v/v streptavidin-peroxidase (Sigma) was added, the plates were incubated for 30 min, and the wells were washed again.

The colorimetric reaction was developed with a TMB (3,3',5,5'-tetramethylbenzidine) substrate solution for 15 min at room temperature. The reaction was stopped by the addition of 100 μl of 2 N sulfuric acid, and absorbance was read at 450 nm using an automated microplate reader (Benchmark; Bio-Rad Laboratories, Hercules, CA, USA). All assays were carried out in duplicate. Positive cutoff values were determined from the mean plus two standard deviations of the absorbance values of the 38 healthy controls.

### ELISA inhibition test

The specificity of IgE binding to the potato extracts was tested and the allergenic potency of the GM and wild-type potato extracts was analyzed by a competitive ELISA inhibition test. Sera from four patients with high levels of specific IgE binding were pooled, preincubated overnight at 4°C with five concentrations (1–100 μg/ml) of either *Dermatophagoides pteronyssinus *or extract from GM or wild-type potatoes, and then incubated for 12 h in microtiter plates coated with extracts from the two types of potato. The same steps were followed as in the ELISA. As a control, samples were preincubated with equal volumes of PBS (pH 7.5) instead of house dust mite or potato extracts. The percentage of inhibition of serum IgE binding was expressed as: 100 - (absorbance of the samples preincubated with allergens/absorbance of the samples preincubated with PBS) × 100.

### SDS-PAGE and immunoblot analysis

SDS-PAGE and immunoblot analysis were carried out under reducing conditions according to previously described methods [13]. Extracts from the two potatoes were mixed with sample buffer (Tris-HCl 31 mmol/l, 10% glycerol, 1% SDS, 0.0025% bromophenol blue, 2.5% β-mercaptoethanol, pH 6.8) and heated in boiling water for 5 min. Standard markers (4–250 kDa; Novex, San Diego, CA, USA) and the extracts were loaded on a 4–20% Tris-glycine gel (Novex). Electrophoresis was done with a Novex X cell™ Mini cell for 90 min at 125 V. The gel was fixed and stained with Coomassie brilliant blue. For immunoblotting, proteins were transferred onto a polyvinylidene difluoride membrane (PVDF; Millipore Co., Bedford, MA, USA) in transfer buffer (Tris-base 25 mmol/l, glycine 193 mmol/l, and methanol 20%) using a Bio-Rad transfer apparatus set at 200 mA for 90 min. The blotted PVDF membrane was sliced into 4-mm widths and then incubated in 5% skim milk in Tris-buffered saline (TBS)-Tween (TBST) for 1 h to block nonspecific binding to the membranes. Each membrane slice was incubated overnight at 4°C with patient or control sera diluted 1:2 v/v with 3% skim milk in TBST, and then washed with 0.1% TBST for 30 min. Subsequently, the membranes were incubated with goat anti-human IgE conjugated with alkaline phosphatase (Sigma) for 1 h at room temperature, washed with TBST, and then developed with BCIP/NBT alkaline phosphatase substrate (Sigma).

### Effect of simulated gastric and intestinal fluid on the IgE-binding components

Crude extracts were prepared from GM and wild-type potatoes and then heated at 100°C for 5 min. The intrinsic digestibility of the extracts and the digestibility of extracts preheated and incubated in SGF or SIF were examined as previously described [14]. Briefly, SGF digestibility was analyzed by dissolving 680 μg of naïve crude extract or preheated extract in 200 μl of prewarmed 100 mmol HCl/l (pH 1.2) and 30 mmol NaCl/l containing 0.32% (w/v) pepsin A (Sigma). Digestion was conducted at 37°C with continuous shaking, and aliquots of the digested solution (20 μl) were withdrawn at 0, 0.5, and 60 min. These aliquots were quickly mixed with 26 μl of sample buffer (containing 2.5% 2-mercaptoethanol and 1% SDS) and 6.0 μl of Na_2_CO_3 _(200 mmol/l). The mixture was then boiled for 5 min and stored at -20°C until further analysis. To evaluate the effects of SIF, 680 μg of the naïve crude extract or the preheated extract were dissolved in 260 μl of prewarmed intestinal control solution (0.05 M KH_2_SO_4_, pH 6.8) containing 1.0% (w/v) pancreatin (pancreatin USP; Sigma). This solution was incubated at 37°C with continuous shaking. Aliquots (26 μl) were withdrawn at 1, 90, and 240 min, mixed with 26 μl of sample buffer (containing 2.5% 2-mercaptoethanol and 1% SDS), and then boiled for 5 min. SDS-PAGE (12%) and IgE-immunoblot analysis were then carried out as described above.

## Results

### Allergy skin-prick test and specific IgE to wild-type and GM potatoes

**Figure 1:**
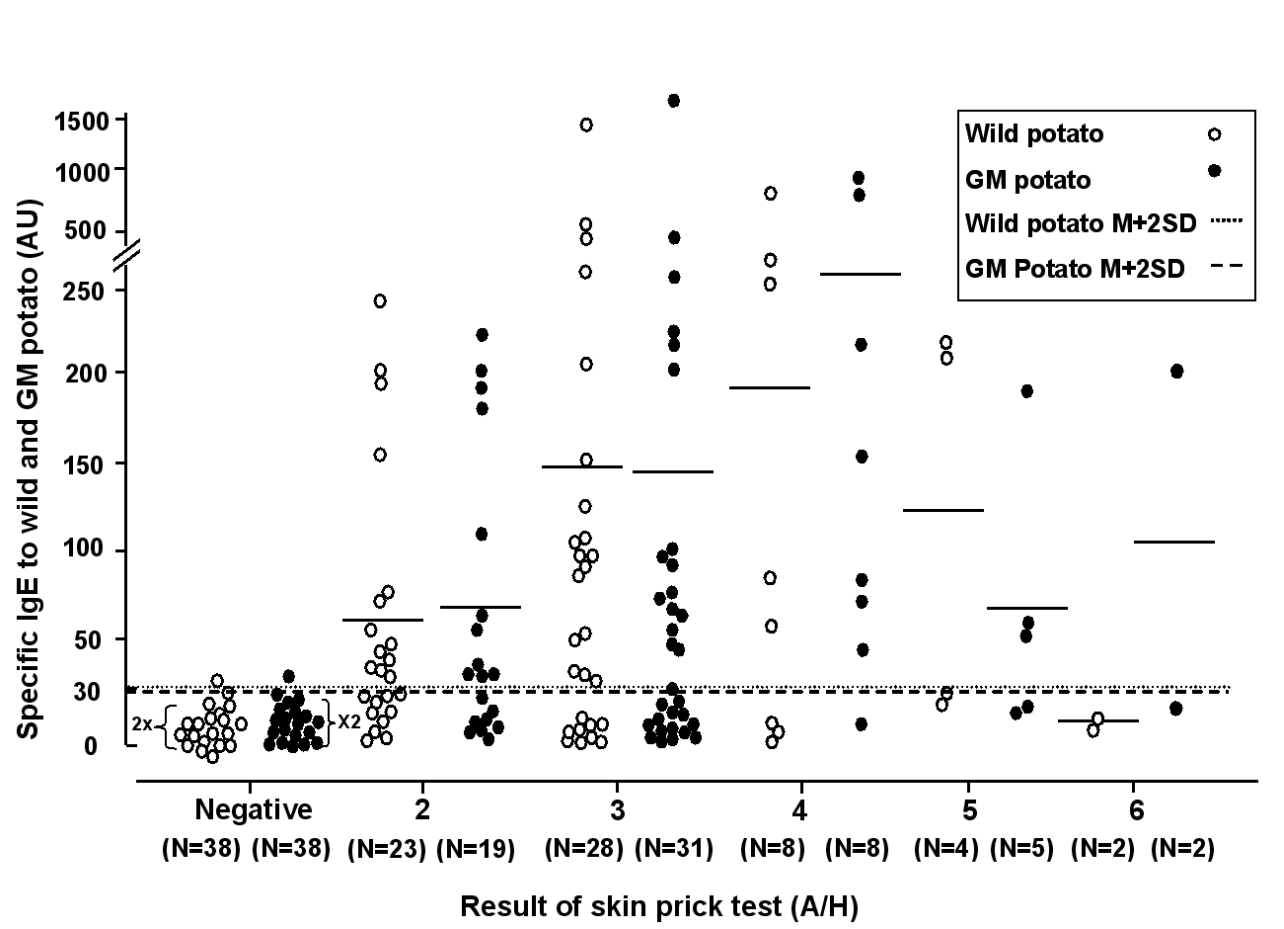
Specific IgE binding to extracts of wild-type and GM potatoes, as determined by ELISA, *vs*. the A/H ratio of the potato. A/H: the ratio of the size of the wheal induced by allergen on allergic skin-prick test to that induced by histamine. N: number of subjects in each response group according to the skin-prick test using potato extract. Positive cutoff values were determined from the mean plus two standard deviations (M + 2SD) of the absorbance values of the 38 healthy controls.

During the one-year study, 108 (5.7%) of the 1886 patients had an A/H score >2+ in response to the skin-prick test using extracts from GM and wild-type potatoes. All of the subjects who reacted positively to the GM potato also had positive responses to the wild-type potato, as seen in Table 2. House-dust mites are the most common aeroallergen in Korea, followed by weed, tree, and grass pollens [15], but, based on allergy skin-prick testing, no specific antigen showing cross-reactivity with potato proteins has been identified. ELISA using sera from subjects with a positive skin prick test showed that the prevalence of specific IgE to extracts of wild-type and GM potatoes ranged from 0–88%, with 57 and 58% (13 of 23 and 11 of 19, respectively) having a 2+ reaction, 64 and 58% (18 of 28 and 18 of 31, respectively) with a 3+ reaction, 63 and 88% (5 of 8 and 7 of 8, respectively) having a 4+ reaction, and 50 and 60% (2 of 4 and 1 of 2, respectively) with a 5+ reaction, 0 and 50% (0 and 1 of 2, respectively) with a 6+ reaction. (Table 2, Fig. 1).

**Table 2:** Specific IgE binding, stratified by the results of skin-prick testing, to extracts of wild-type and GM potatoes.

A/H ratio		Wild-type potato					GM potato				
		
		2+	3+	4+	5+	6+	2+	3+	4+	5+	6+
Skin-prick test result n (%)		38 (35.2)	48 (44.5)	12 (11.1)	7 (6.4)	3 (2.8)	36 (33.3)	51 (47.2)	13 (12)	6 (5.6)	2 (1.9)
sIgE (%) to	Wild	13/23 (57)	18/28 (64)	5/8 (63)	2/4 (50)	0/2 (0)	8/19 (42)	17/31 (55)	6/8 (75)	1/5 (20)	0/2 (0)
	GM	10/23 (44)	16/28 (57)	5/8 (63)	1/4 (25)	0/2 (0)	11/19 (58)	18/31 (58)	7/8 (88)	3/5 (60)	1/2 (50)

Positive skin-prick test response (≥2+): 108/1886 for wild-type and genetically modified (GM) potato. A/H ratio: the ratio of the size of the wheal induced by allergen on allergic skin-prick test to that induced by histamine; N: number of subjects positive to skin-prick test for wild-type and GM potato; sIgE: prevalence of serum specific IgE antibody to wild-type and GM potato (number of subjects with positive serum specific IgE/number of subjects tested in each response group according to the skin-prick test using potato extract).

### ELISA inhibition test

**Figure 2:**
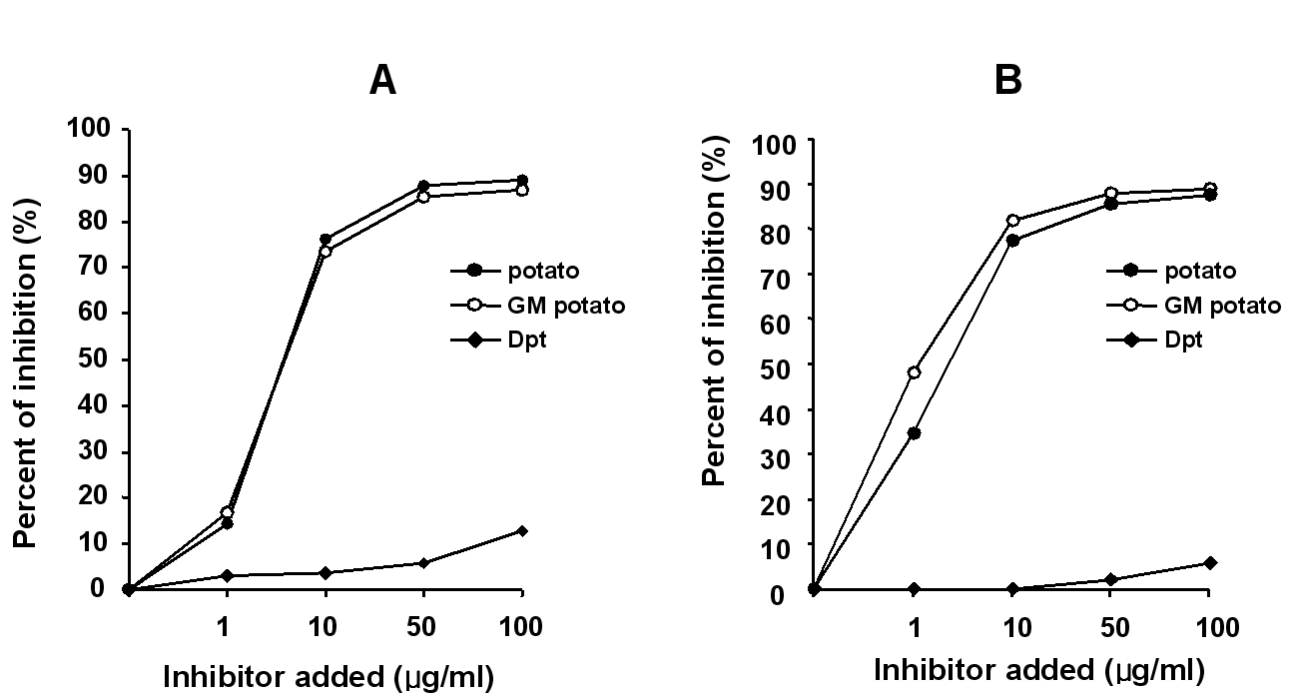
Percent inhibition of IgE-ELISA by the addition of: extract from wild-type potato (●), GM potato extract (○), and *Dermatophagoides pteronyssinus *(◆). Sera from patients sensitized to potatoes were used. A: wild-type potato; B: GM potato.

The ELISA inhibition test showed significant dose-dependent inhibition for wild-type and GM potato extracts. In addition, the two extracts had similar potencies. By contrast, minimal inhibition was noted using *D. pteronyssinus*. Figure 2 shows the ELISA inhibition test by the addition of extracts from wild-type and GM potato and *Dermatophagoides pteronyssinus*. The same pooled sera from patients sensitized to potatoes were used (A: wild-type potato; B: GM potato).

### SDS-PAGE and IgE-immunoblot analysis

**Figure 3:**
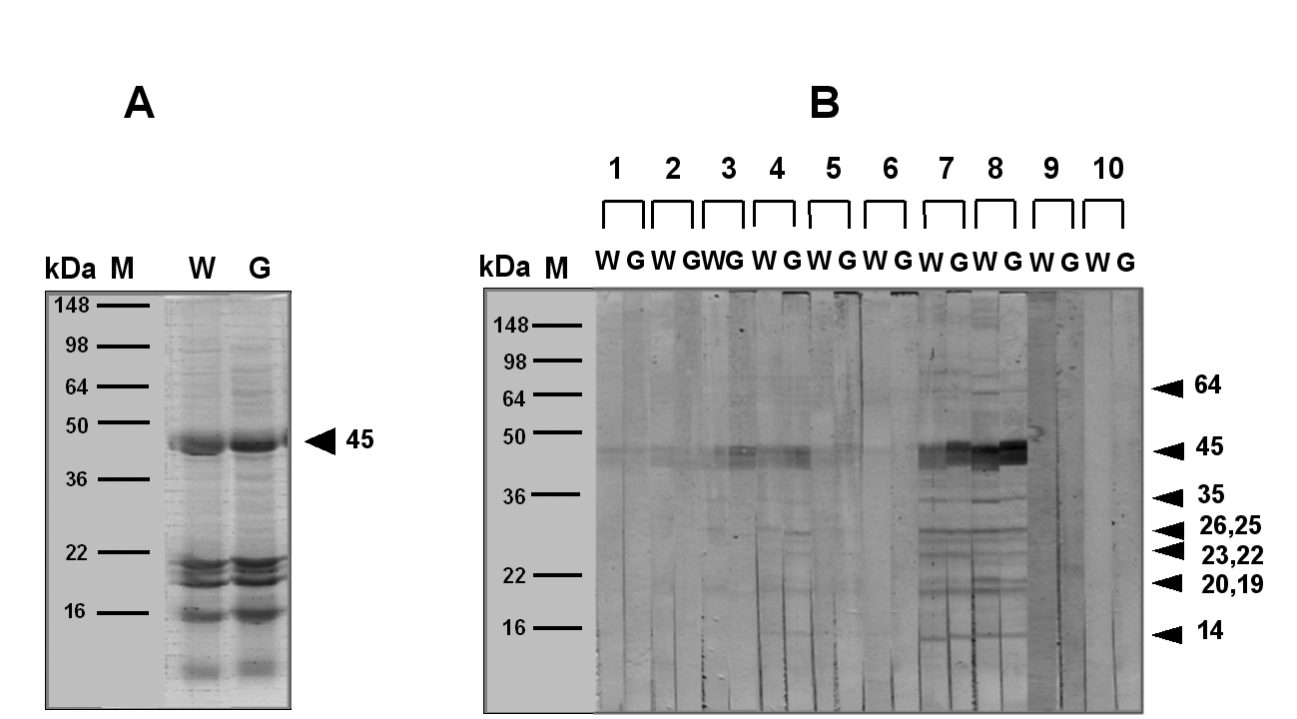
SDS-PAGE of proteins (A) and IgE-binding components (B) present in wild-type and GM potato extracts, as measured in sensitized patients. W: wild-type potato extracts; G: GM potato extracts. M: marker; lanes 1–8: sensitized subjects; lane 9: normal control; lane 10: buffer control.

The IgE-binding components within the wild-type and GM potatoes were compared using the sera of eight individuals with high specific IgE levels and sera pooled from the controls. The latter was derived from 10 patients who responded negatively to the two potato extracts on the skin-prick test. Three components (45, 34, and 26 kDa) were noted in >50% and 11 components (78, 72, 64, 36, 35, 25, 23, 22, 20, 19, and 14 kDa) in 33% of patients sensitized to wild-type potato. One component (45 kDa) was noted in 88% and eight components (78, 64, 35, 26, 25, 23, 22, and 14 kDa) in <50% of patients sensitized to GM potato. Thus, the 45-kDa band present in serum was the most frequently bound (>80%) by extracts from wild-type and GM potatoes (Fig. 3).

### Effect of digestive enzymes on extracts of GM and wild-type potatoes

**Figure 4:**
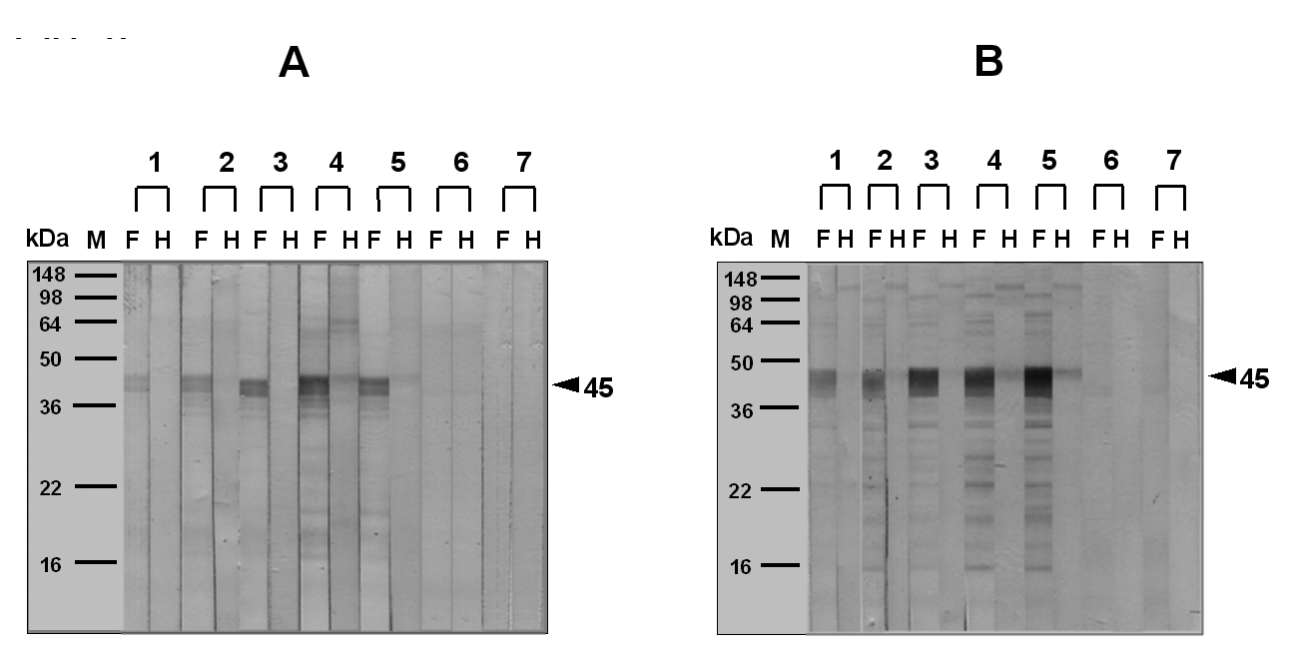
Effect of boiling on wild-type (A) and GM (B) potato allergenicity, analyzed by IgE-immunoblotting. F: Fresh potato extract; H: heated extract. Lanes 1–5: sensitized subjects; lanes 6 and 7: normal control; lane 8: buffer control.

**Figure 5:**
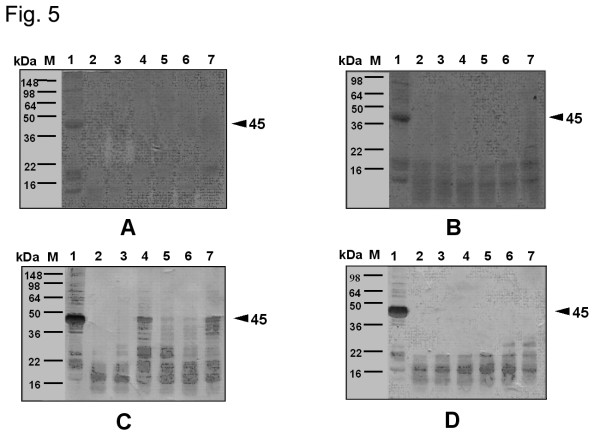
Effect of simulated gastric fluid (SGF) treatment on extracts from wild-type and GM potatoes, as determined by SDS-PAGE (A, B), and on IgE-binding components present in the extracts (C, D). A, C: extracts from wild-type potato; B, D: extracts from GM potato; M: marker; lanes 1–7: incubation for 0 s, 30 s, 1 min, 10 min, 30 min, 1 h, and 1.5 h with sera pooled from five patients. pH of SGF: ca. 1.2.

**Figure 6:**
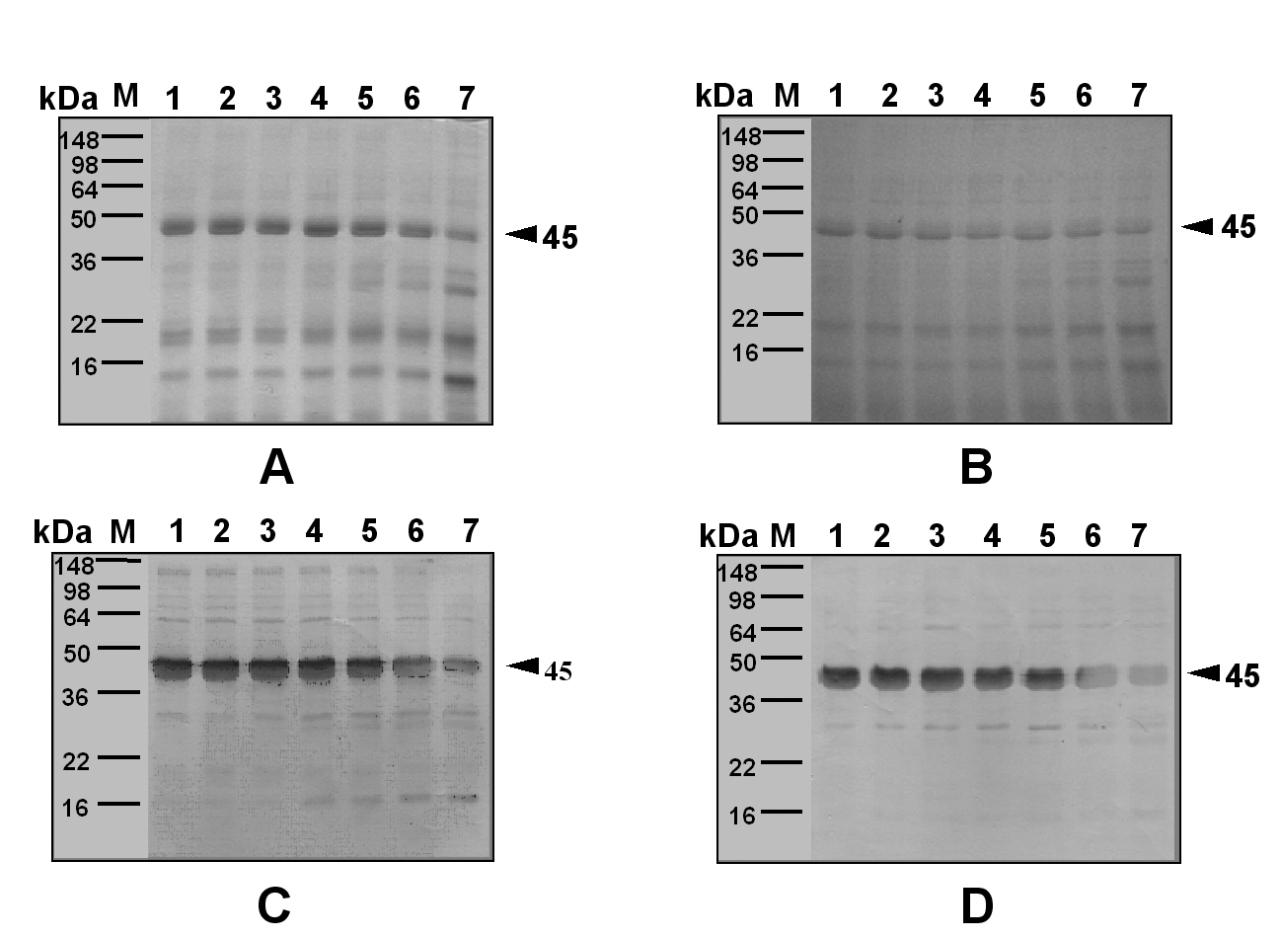
Effect of simulated intestinal fluid (SIF) treatment on extracts from wild-type and GM potatoes, as determined by SDS-PAGE (A, B), and on IgE-binding components present in the extracts (C, D). A, C: extracts from wild-type potato; B, D: extracts from GM potato; M: marker; lanes 1–7: incubation for 0 s, 30 s, 1 min, 10 min, 30 min, 1 h, and 4 h. pH of SIF: ca. 7.5.

Figures 4, 5, and 6 demonstrate the changes both in the proteins, as seen on SDS-PAGE, and in the IgE-binding components of extracts from wild-type and GM potatoes after SGF or SIF treatments in the presence or absence of heating. A combination of SGF and heat treatment resulted in the disappearance of the protein bands and the IgE-binding components in extracts from wild-type and GM potatoes, while minimal changes were noted with SIF treatment alone.

## Discussion

The results of this study demonstrate that the prevalence of positive skin-prick tests to extracts from wild-type and GM potatoes was 5.7% in our population of adult allergy patients. Serum-specific IgE antibodies were detected among the positive responders. The 45-kDa allergenic band was the most frequently bound (>80%) by the two types of extract, and ELISA inhibition studies suggested that they had similar potencies. Recent investigations to evaluate the allergic risks of GM corn and soybean demonstrated that allergenicity did not increase after genetic manipulation of wild-type corn [11] and soybean [9]. Similarly, our study showed that the genetic manipulation of potatoes, which are one of the most common sources of food allergens in Korea, did not enhance their allergenic risk, as evaluated using *in vivo *and *in vitro *methods.

GM crops currently on the market have been thoroughly assessed for safety according to the guidelines developed by the World Health Organization [16] and the Food and Agriculture Organization of the United Nations [17]. In addition, the potential allergenicity of newly introduced proteins must be assessed in all foods produced through agricultural biotechnology, and the FAO [18] and WHO have developed a rigorous approach for this assessment. The *NPTII *gene introduced in the potato used in this study encodes an enzyme that confers resistance to aminoglycoside antibiotics and was isolated from the prokaryotic transposon Tn5 [19]. The *PAT *gene was obtained from the aerobic soil bacterium *Streptomyces viridochromogenes*. If the gene source is bacterial, specific and targeted serum screenings are not necessary because bacterial proteins are rarely allergenic, due to the low exposure levels and lack of allergic sensitization to these proteins [8]. Furthermore, previous studies have confirmed that ingestion of genetically engineered plants expressing NPTII does not pose any safety concerns [20,21]. Herouet et al. [22] found that PAT proteins do not possess the characteristics associated with food toxins or allergens, i.e., they have no sequence homology with any known allergens or toxins. These findings suggest that an increase in the allergenic risk of a GM potato is unlikely.

A few studies have identified IgE-binding components within potato extracts. Wahl et al. [23] reported four (16, 30, 45, and 65) IgE-binding components, which were detected by immunoblotting using sera from 12 patients with IgE-mediated hypersensitivity reactions to potatoes. Four major potato allergens, Sol t 1 (43 kDa), 2 (21 kDa), 3 (21 kDa), and 4 (16 kDa), were identified in children [24,25]. Furthermore, several studies have shown that allergy to natural rubber latex is associated with cross-reactivity to potatoes and tomatoes [7,26]. In our study of the Korean population, only one allergenic protein (45 kDa) could be identified as the major allergen, and it was present in extracts from wild-type and GM potatoes. Moreover, this protein may be the same one identified in a previous investigation [23].

Proteolytic stability is a useful criterion in assessing the allergenic potential of food allergens. The FAO/WHO [18] decision-tree approach advocates the use of resistance to proteolysis with pepsin as a comparative measure of digestive stability for proteins introduced into food through agricultural biotechnology. In the current study, we demonstrated that SGF and heat treatment substantially suppressed the activity of IgE-binding components in wild-type and GM potato extracts, while minimal changes were noted with SIF treatment alone.

## Conclusion

We report that the sensitization rate of patients to wild-type and GM potato extracts was 5.7%, and the prevalence of specific IgE to these extracts in the sera of subjects with a positive skin-prick test was similar. An ELISA inhibition test showed significant dose-dependent inhibition by GM and wild-type potato extracts, and the two extracts had similar potencies. A 45-kDa band was identified as the most frequently bound by both extracts. Our results strongly suggest that genetic manipulation of potatoes using antibiotic resistance and herbicide tolerance genes does not increase their allergenic risk.

## Abbreviations

GM = genetically modified

ELISA = enzyme linked immunosorbent assay

SDS-PAGE = sodium dodecyl sulfate-polyacrylamide gel electrophoresis

NPTII = neomycin phosphotransferase II

PAT = phosphinothricin acetyltransferase

SGF = simulated gastric fluid

SIF = simulated intestinal fluid

## Authors' contributions

SKL: ideas, study design and writing

YMY: ideas and writing

SHY: laboratory work

BOL: preparation of GMO and non -GMO potato

SHK: study design and laboratory work

HSP: ideas, study design, laboratory work and writing
